# Get It While It’s Hot: A Peak-First Bias in Self-Generated Choice Order in Rhesus Macaques

**DOI:** 10.1371/journal.pone.0083814

**Published:** 2013-12-23

**Authors:** Kanghoon Jung, Jerald D. Kralik

**Affiliations:** Department of Psychological and Brain Sciences, Dartmouth College, Hanover, New Hampshire, United States of America; Institut Pluridisciplinaire Hubert Curien, France

## Abstract

Animals typically must make a number of successive choices to achieve a goal: e.g., eating multiple food items before becoming satiated. However, it is unclear whether choosing the best first or saving the best for last represents the best choice strategy to maximize overall reward. Specifically, since outcomes can be evaluated prospectively (with future rewards discounted and more immediate rewards preferred) or retrospectively (with prior rewards discounted and more recent rewards preferred), the conditions under which each are used remains unclear. On the one hand, humans and non-human animals clearly discount future reward, preferring immediate rewards to delayed ones, suggesting prospective evaluation; on the other hand, it has also been shown that a sequence that ends well, i.e., with the best event or item last, is often preferred, suggesting retrospective evaluation. Here we hypothesized that when individuals are allowed to build the sequence themselves they are more likely to evaluate each item individually and therefore build a sequence using prospective evaluation. We examined the relationship between self-generated choice order and preference in rhesus monkeys in two experiments in which the distinctiveness of options were relatively high and low, respectively. We observed a positive linear relationship between choice order and preference among highly distinct options, indicating that the rhesus monkeys chose their preferred food first: *i.e.*, a peak-*first* order preference. Overall, choice order depended on the degree of relative preference among alternatives and a peak-first bias, providing evidence for prospective evaluation when choice order is self-generated.

## Introduction

Animals live in the present but benefit from remembering the past and predicting the future. For goal-directed behavior, it has long been appreciated that many animals, including humans, consider future events, but discount their value based on the delay to receiving them, indicating that an immediate reward is more valuable than the same amount of reward in the future [1]–[7]. At the same time, when remembering past events, animals tend to remember the most recent ones best—i.e., there is a recency effect—and thus they also discount the value of past events [8]. With respect to outcome evaluation, this would mean that the recent past reward is more valuable than the same amount of reward in the more distant past.

In fact, this retrospective evaluation process has been well-documented in people, leading to what has been called a *peak-end* rule in which people judge previous events by averaging the peaks (i.e., the most salient, highest intensity moments) and the end [1]–[7], [9]–[15]. In particular, the peak-end rule implies that individuals who follow it will prefer events that end well. That is, because the average is higher when the highest peak is at the end, people will have a *peak-end bias*. Indeed, there are numerous examples in everyday life that appear to reflect this preference: from dessert at the end of meals to the climax of stories or performances normally occurring at the end.

Thus, outcome evaluation appears to be a decreasing function of distance in time from the present to both the future and the past. However, the conditions under which individuals use prospective or retrospective evaluation to guide behavior remain unclear. We hypothesize that the type of evaluation process evoked depends in part on the extent to which a series of events are organized into a unit or episode. When individual events are seen as parts of a larger episode, the evaluation process may be conducted on the episode as a whole once it is completed. In this case, memory is needed, and retrospective evaluation would occur. If, however, the events are seen as separate entities, prospective evaluation would take place, with each event being processed individually in order to maximize immediate reward.

As a test of this hypothesis, we conducted a study with rhesus monkeys, which have proven to be an excellent primate model of human decision-making; and at the same time help delineate the evolutionary origins of these evaluation processes. It had previously been shown that when receiving a sequence of qualitatively distinct rewards, rhesus monkeys did not exhibit a clear peak-end bias, and if anything, appeared to show a peak-*first* one [1]–[7], [16]. We suggest that with qualitatively distinct items, subjects may generally evaluate them separately, and thus use a prospective evaluation process.

Following the logic of items being evaluated prospectively if considered individually, we hypothesized that if the monkeys were offered four qualitatively distinctive food items, which they could obtain in any sequence and were allowed to generate the sequence themselves, they would evaluate each item in the sequence individually, leading to prospective evaluation and a preference for a peak-*first* sequence. Thus, for the current study, we asked, if given a sequence of choices without replacement, in which all items are initially available and an individual must select them in their preferred order, which evaluation process would be used? Subjects who determined the sequence retrospectively should have a peak-*end* bias and should choose in the *opposite* order of their preference, saving the best for last, whereas those who chose prospectively, should have a peak-*first* bias and should choose in the order of their preference, selecting their favorite first and so on. We found that all four monkeys exhibited strong peak-*first* order preferences, but only when there were large enough differences in preferences among the food items.

## Methods

### Ethics statement

Animal care and use complied with all current laws, regulations, policies, and guidelines of the United States, the United States Department of Agriculture (USDA), the Public Health Service (PHS), and all procedures were approved by the Institutional Animal Care and Use Committee (IACUC) of Dartmouth College.

### Subjects

Four male rhesus macaques (*Macaca mulatta*) participated in Experiments 1 and 2: Hamlet, Caesar, Puck, and Titus. Hamlet, Puck, and Titus. The average age of the monkeys was 8.75±0.48 (mean ± s.e.m). They were housed in 32×27×68 (width x depth x height) inch cages (Allentown Inc., Allentown, NJ) in a homeroom with automatically regulated temperature, ventilation, humidity, and lighting (14 and 10 hours for light and dark cycles, respectively, with lights on at 6 am). The monkeys were intermittently housed in pairs and individually: at times when they engaged in confrontations, which is normal periodic behavior in young rhesus macaque males of similar size and temperament [8], [17], the two monkeys were separated and individually-housed for their safety.

The Center for Comparative Medicine and Research (CCMR) at Dartmouth maintains a full-time animal care and veterinary staff that monitors the monkeys’ daily health and well-being. The monkeys were maintained at approximately 95% of their *ad libitum* weights to ensure sufficient motivation and good health, and their diet consisted of primate chow (no. 5038, PMI Feeds Inc., St Louis, Missouri, U.S.A.), supplemented with fresh fruit and vegetables, as well as various treats that included peanuts, cereal, and dried fruits (*e.g.*, raisins, banana).

To allow for continued social stimulation, the subjects had direct visual contact with the other monkeys in the colony, the animal care staff, and experimenters. When pair-housed, they had direct physical contact with each other, and also when individually-housed, through a mesh grading divider between their cages. In addition, environmental enrichment included two or more enrichment items in their home cages at all times, daily playing of radio or videos in the room (the latter via a monitor mounted in view of all individuals), and regular access to a larger enrichment cage (68×38×72 inch) in an adjacent room. The monkeys were brought to the testing room in custom-made chairs. The chairs were used (a) to minimize disruption in the test subjects’ daily routines, given that they were already acclimated to them from previous experiments; (b) to have precise control over the experimental testing conditions; and (c) to obtain clear, unbiased choice responses, with tray compartments (described below) at fixed positions relative to the monkey on every trial.

### Materials

Each monkey sat across from the experimenter in the chair. The chair loosely restrained the left arm of the monkey while allowing free movement of the right arm. An opaque plastic divider separated the experimenter and the monkey, thus preventing the monkey from seeing the face and upper body of the experimenter. An opening at the bottom of the divider allowed the experimenter to present a transparent plastic food tray to the monkey. The tray contained four separate compartments, which we labeled according to their position relative to the experimenter: left (L), middle left (ML), middle right (MR), and right (R). On a given trial, food was placed on a circular platform in a compartment so that the monkey could clearly see and easily select his choice of food item. Each compartment was covered by a transparent lid, which the monkey had to lift to gain access to a food item. The food items in Experiment 1 consisted of four different types of food, with all being generally yellow in color: shelled peanut halves (PN), yellow BioServ® Fruity Gems (FG), BioServ® banana-flavored dustless precision pellets (PL), and rice krispies®, or krisps (KR). The food items in Experiment 2 consisted of four differently colored (red, orange, green, and purple) BioServ® Fruity Gems (FG).

### Procedure

Both Experiments 1 and 2 consisted of a two-condition interleaved paradigm (described below) that studied ordered choice among four food items without replacement (Condition 1) and binary choice between two food items (Condition 2). Four different types of food (PN, FG, PL, and KR) for Experiment 1 and four differently colored fruity gems for Experiment 2 were used. Because the binary choice condition allowed only one selection per trial, and the loss of the item not selected, we reasoned that choice in this condition would reveal preference. In the ordered choice condition without replacement, the monkeys would obtain all four food items regardless of order; and thus, selection order might or might not reflect preference. For example, an individual might select his favorite items first, or save them for last. The experiments, therefore, examined the specific relationship between choice order and preference.

The conditions were interleaved in that each test session consisted of 1 trial of Condition 1 (i.e., four selections without replacement to the monkey) and 12 trials of Condition 2 (i.e., all six combinations of the four items multiplied by two presentation positions). We interleaved the conditions to make sure choice order and preferences remained stable; and we conducted the ordered choice condition first to provide the monkeys with experience of all four food items before conducting the binary choice preference tests.

**Figure 1:**
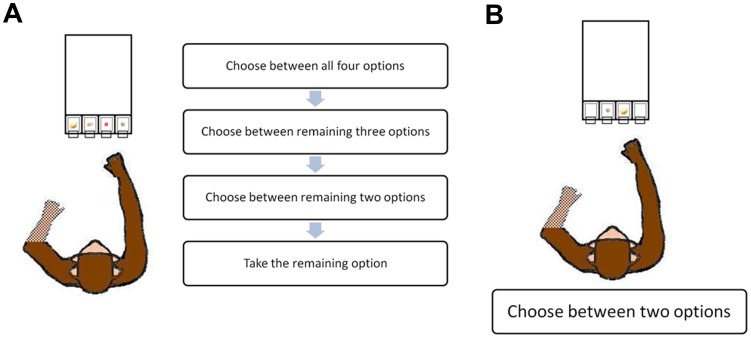
Diagrams of the experimental procedures used in the two conditions. (A) Diagram for choices among four food items without replacement used in Condition 1. (B) Diagram for the binary choice paradigm used in Condition 2.

In Condition 1, the ordered choice condition, a different food item was placed into each of the four compartments (Figure 1A). The location of food items by compartment was pseudorandomly assigned across sessions. To begin the trial, the experimenter slid the tray on the table towards the monkey to a position just out of reach, and paused for approximately three seconds for the monkey to observe the items. The tray was then moved toward the monkey to allow him to make a selection by lifting the lid of the compartment and taking the food item contained within. The experimenter then withdrew the tray to a position underneath the divider that nonetheless remained in continuous view of the monkey, so the monkey could see the remaining food items and that nothing otherwise had changed. After approximately three seconds to allow the monkey to eat the selected food item, the tray was again slid forward following the same procedure. This was repeated until all four food items were taken by the monkey. The selection of all four food items was considered to be one complete trial.

To measure food preferences, in Condition 2 we used the binary choice paradigm, in which two different food items were placed in the two middle containers (ML and MR), with one food item per container (Figure 1B). To begin the trial, the experimenter presented the two food items by (a) moving the apparatus towards the monkey to a position just out of reach, to allow the monkey to observe the items for approximately three seconds, and then (b) further towards the monkey to allow him to make a selection by both lifting the lid of the compartment and taking the food item contained within. The experimenter then withdrew the apparatus and began a new trial with a new permutation of two food items. The arrangement of the food items by compartment was pseudorandom such that no permutation was repeated within a session. We conducted four sessions per day on six different days, for a total of 24 sessions (with each session consisting of one trial of Condition 1, which was four selections without replacement, and 12 trials of Condition 2).

Once the data was collected, for Condition 1, we calculated the choice rate of each item for a given choice order. The overall individual's choice order with respect to different items was determined by selecting an item that provides the highest choice rate for a given order. For Condition 2, we calculated the winning percentage of each food item, the percentage of winning in the binary choice paradigm. Then we obtained the rank order of individual preference by ranking individual's winning percentages of food items.

## Results

### Experiment 1

**Figure 2:**
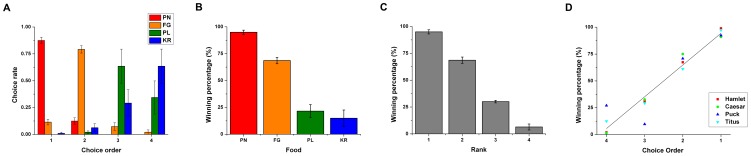
Choice order and preference in Experiment 1. (A) The choice rate of food items for a given choice order averaged over all monkeys in Condition 1; peanut half (PN), yellow fruity gem (FG), pellet (PL), and krisp (KR). (B) The winning percentage of different food items in Condition 2 (binary choice). (C) The winning percentage of different food items in Condition 2 (binary choice) sorted by individual rank order, showing a significant preference towards the favorite options. (D) The relationship between choice order and winning percentage reflecting the degree of preference among alternatives**.** Error bars depict standard error of the mean (SEM).

In Condition 1, we tested the order in which the monkeys chose a food item among the four different food items without replacement. Overall, the monkeys predominantly chose the peanut half (PN; mean ± s.e.m = 87.5±4.2%), fruity gem (FG; 79.2±4.8%), pellet (PL; 63.5±22.4%), and rice krisp (KR; 63.5±22.5%) for their first, second, third, and fourth choices, respectively (Figure 2A). There were significant differences in choice rate for the order of every food item (One-way ANOVA, *F*(3,12)  = 408.0, *p*<0.001 for PN; *F*(3,12)  = 148.8, *p*<0.001 for FG; *F*(3,12)  = 7.4, *p*<0.01 for PL; *F*(3,12)  = 7.7, *p*<0.01 for KR). This indicates that the four monkeys showed a similar choice order for each food item. Individually, all four monkeys exhibited significant choice orders for the different food items across sessions (Chi-squared test, Hamlet: χ^2^(9)  = 204.0, *p*<0.001; Caesar: χ^2^(9)  = 192.0, *p*<0.001; Puck: χ^2^(9)  = 110.0, *p*<0.001; Titus: χ^2^(9)  = 158.7, *p*<0.001 ), indicating a stable choice order for each food item across sessions.

For Condition 2, we calculated the winning percentage of each food item using a binary choice paradigm that presented all possible combinations of two food items in order to measure preference between food items. Figure 2B shows that the peanut half had the highest winning percentage (95.0±2.0%), followed by fruity gem (68.6±2.9%), then pellet (21.5±6.1%), then krisp (14.9±7.6%). There was a significant difference of winning percentage between food items (One-way ANOVA, *F*(3,12)  = 54.8, *p*<0.001), indicating general food preferences in the monkeys.

Individually, all four monkey showed significant preferences toward an option among the four alternatives in Condition 2 (Chi-squared test, Hamlet: χ^2^(3)  = 154.9, *p*<0.001; Caesar: χ^2^(3)  = 143.1, *p*<0.001; Puck: χ^2^(3)  = 126.0, *p*<0.001; Titus: χ^2^(3)  = 120.8, *p*<0.001). The food preferences were the same for three of the monkeys (Hamlet, Caesar and Puck, from most to least preferred: PN, FG, PL, KR), and somewhat similar for the other monkey (with the first- and second-most preferred being the same; from most to least preferred, Titus: PN, FG, KR, PL). All four monkeys preferred the peanut half most, then the fruity gem. Overall, there were strong food preferences for all individuals and small individual differences in less-preferred items.

Because all four monkeys showed the greatest preference for peanut halves and the second-greatest preference for fruity gems it indicates general preferences for these food items. This general preference suggests there are particular factors, such as caloric value, that may underlie preference. Thus, we tested whether the winning percentage was correlated with the caloric values of the food items (i.e., calorie per item divided by total calories of all four food items multiplied by 100): peanut half (66.9%); fruity gem (25.5%), pellet (5%), krisp (2.6%). The winning percentage for each food item was indeed significantly correlated with their caloric values (Pearson-*r* = 0.92, *n* = 16, *p*<0.001).

Although the individual monkeys exhibited similar preferences among food items, individual preference does not necessarily correspond to group preference (especially as seen in Experiment 2). Thus, we defined rank as the order of winning percentage within individuals. Then, we compared the winning percentage with respect to rank to quantify the extent of preference toward an individual's favorite options (Figure 2C). There was a significant difference of winning percentage between ranks (One-way ANOVA, *F*(3,12)  = 287.1, *p*<0.001), confirming that individual preference among ranks was significantly biased toward the favorite options.

Comparing the two conditions, we found a direct linear relationship between choice order and the winning percentage of food items (Overall: slope  =  29.5, Adj. R-Square  =  0.918, *p*<0.001) (Figure 2D). Regarding choice order in Condition 1, three of the four monkeys chose items in order of their preferences from Condition 2, with the most preferred item chosen first (Hamlet: slope  =  32.8, Adj. R-Square  =  0.998, *p*<0.001; Caesar: slope  =  31.1, Adj. R-Square  =  0.961, *p*<0.05; Titus: slope  =  28.6, Adj. R-Square  =  0.964, *p*<0.05); the fourth monkey, Puck, chose his first and second preferred items in order, but then flipped the choice order for the third and fourth preferred items (Puck: slope  =  25.7, Adj. R-Square  =  0.63, *p* = 0.13). It is unclear why Puck tended to select the fourth preferred item (pellet) over the third (krisp) when choosing between them in the choice order condition, but selecting the third over the fourth in the binary choice condition. Although the difference in winning percentage between the third and fourth preferred items (17.4%) was less than the overall average difference (29.5%) or Puck’s average difference (27.6%), it, however, was not the smallest difference among the preferences (which was the difference between Caesar’s first and second preferred items: 16%).

Overall, the monkeys exhibited a general peak-first bias, with at least the first and second favorites being selected in order.

### Experiment 2

To help delineate the factors underlying preference and choice order, Experiment 2 tested color, which is considered a salient cue to primates with trichromatic color vision, such as rhesus monkeys [1]–[7], [9]–[15], [18]–[22]. The experiment repeated the two conditions of Experiment 1 with fruity gems of four different colors—red, green, orange, and purple—but the same nutritional value, to test (a) whether there was a general choice order with respect to color, (b) whether there were individual differences in color preference and (c) if (b), whether there was a relationship between choice order and color preferences.

**Figure 3:**
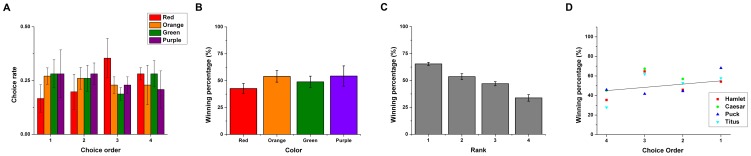
Choice order and preference in Experiment 2. (A) The choice order matrix representing the choice percentages of different colored items for given choice order averaged across all monkeys in Condition 1. (B) The winning percentage of different color items in Condition 2 (binary choice). (C) The winning percentage of different color items in Condition 2 (binary choice) sorted by individual rank order, revealing a significant individual preference among the colors. (D) The relationship between choice order and winning percentage that reflects the degree of preference among alternatives**.** Error bars depict standard error of the mean (SEM).

In Condition 1, we tested the order in which the monkey chose the four differently colored but otherwise identical food items (fruity gems). The monkeys chose green (mean ± s.e.m = 28.1±6.5%) and purple (28.1±11.1%) most for their first choice in a two-way tie, purple most for their second choice (28.1±5.0%), red most for their third choice (35.4±9.2%), and red (28.1±2.8%) and green (28.1±6.1%) most for their fourth choice in a two-way tie (Figure 3A). There were no significant differences in choice rate for the order with respect to each color (One-way ANOVA, *F*(3,12)  = 2.9, *p* = 0.079 for red; *F*(3,12)  = 0.27, *p* = 0.848 for orange; *F*(3,12)  = 1.3, *p* = 0.330 for green; *F*(3,12)  = 0.46, *p* = 0.715 for purple). This indicates that there was no generally preferred order with respect to color among the monkeys. Individually, three of the four monkeys exhibited no significant choice order for the different colors across sessions (Chi-squared test; Hamlet: χ^2^(9)  = 5.3, *p* = 0.810; Caesar: χ^2^(9)  = 12.5, *p* = 0.188; Titus: χ^2^(9)  = 13.6, *p* = 0.138), indicating an unstable choice order with respect to color across sessions. In contrast, one monkey exhibited a significant choice order for color (Chi-squared test, Puck: χ^2^(9)  = 21.0, *p*<0.013).

For Condition 2, we computed the winning percentage of colored items in the binary choices that showed all possible combinations of two colored items. Figure 2B shows that there was no significant difference between winning percentages with respect to color across individuals (One-way ANOVA, *F*(3,12)  = 0.713, *p* = 0.563). Individually, however, all four monkeys had significant color preferences (Chi-squared test, Hamlet: χ^2^(3)  = 13.3, *p*<0.01; Caesar: χ^2^(3)  = 21.6, *p*<0.001; Puck: χ^2^(3)  = 12.8, *p*<0.01; Titus: χ^2^(3)  = 20.1, *p*<0.001 ) (Figure 3C). The color preferences were the same for two of the monkeys (Caesar and Puck, from most to least preferred: purple, green, orange, red), and somewhat similar for the other two monkeys (with the first- and third-most preferred being the same; from most to least preferred, Hamlet: orange, purple, red, green; Titus: orange, green, red, purple). Overall, there were strong color preferences for all individuals, but large individual differences in specific preferences.

Since there were large individual differences in specific color preferences, we defined rank as the order of winning percentage within individuals. Then, we compared the winning percentage with respect to rank to measure the extent of preference toward an individual's favorite option (Figure 3C). There was a significant difference in winning percentage among ranks (One-way ANOVA, *F*(3,12)  = 31.1, *p*<0.001), indicating that individual preference was significantly biased toward the favorite colors.

Comparing the results of Conditions 1 and 2, there was no significant correlation between choice order and winning percentage, indicating that there was no significant relationship between choice order and individual color preference; and thus no preferred color to be chosen first and no least favorite color to be taken last (Overall: slope  =  3.33, Adj. R-Square  =  0.03, *p* = 0.25) (Figure 3D).

Examining choice order in relation to specific color preferences individually, none of the monkeys exhibited a significant tendency to choose the most preferred item (Hamlet: slope  =  3.75, Adj. R-Square  =  –0.27, *p* = 0.61; Caesar: slope  =  5.42, Adj. R-Square  =  –0.21, *p* = 0.56; Puck: slope  =  6.94, Adj. R-Square  =  0.32, *p* = 0.26; Titus: slope  =  6.12, Adj. R-Square  =  0.20, *p* = 0.32). Thus, the monkeys did not exhibit an order preference with respect to color.

### Relative preference in Experiments 1 and 2

Finally, to compare the relative preference among the choice options in Experiments 1 and 2, we calculated the average difference among winning percentages for each monkey in Experiment 1 (i.e., the difference between the first and second highest winning percentages, second and third, third and fourth, and averaging them for each monkey) and compared them to the same in Experiment 2 (overall: Experiment 1, 29.5±1.1%; Experiment 2, 10.5±0.8%). The average difference in relative preference among the food items was significantly different between the two experiments (paired *t*(3)  = 14.7, *p*<0.001). Thus, although significant preferences were found among the food items in both experiments, the relative preference among options was significantly higher in Experiment 1 than in Experiment 2.

## Discussion

In this study, we examined the relationship between choice order and preference in a self-generated choice sequence to help delineate the conditions under which prospective or retrospective evaluation is used to guide goal-directed behavior. In Experiment 1, the binary choice condition enabled us to quantify preferences for the four food items. In the choice order condition, all four monkeys chose the first two items in order of their preference. Three of the four selected all four items in order of preference, while the fourth monkey flipped the choice order with the last two food items. Overall, the monkeys exhibited a strict ‘best to worst’ choice order. Individually, they each exhibited a general peak-first bias in self-generated choice sequence, with at least the first and second favorites being selected in order.

Experiment 2 helped to delineate the factors underlying choice order, and to examine more closely how preference relates to choice order. On the one hand, we found no clear color preferences consistent across all monkeys. Perhaps the only mildly consistent result was that the color red was least preferred by two monkeys and the second least preferred by the other two. Although there is evidence for some aversion to the color red in rhesus macaques [19], it is less clear why it would be so in the food domain [18], [20]–[22]. One possibility is that the red color of the fruity gem may have been too artificial or extreme, signaling something less edible. On the other hand, all four monkeys did exhibit significant individual color preferences. However, the individual color preferences did not influence choice order in three of the four monkeys.

Comparing the results from Experiments 1 and 2, overall, choice order only appeared to be influenced by preferences when there was a sufficiently large difference in relative preference among the choice options. Indeed, even though the relative preference differences in both experiments were significant, we found a significantly larger average difference in relative preference (measured by winning percentage) across the food items in Experiment 1 compared to Experiment 2. Thus, the relationship between relative preference and behavior—in this case choice order—is complex and apparently nonlinear in rhesus macaques. It will be important for future work to further characterize this nonlinear functional relationship to determine when and how relative differences become sufficiently salient to influence choice order [23]–[25]. Other factors related to relative value and choice order should also be systematically studied, including choice set size, given that decisions generally become increasingly difficult with increasing set size [12], [26]–[28].

However, when the relative differences were sufficiently salient, choice order was clear: when allowed to take four food items in any sequence, the monkeys chose the food items in order of preference, starting with their favorite first. In a study in which one choice response was followed by a sequence of outcomes (i.e., an experimenter generated rather than subject generated sequence), rhesus monkey order preferences were less clear, although if anything, they also tended to exhibit a peak-first preference [16].

In contrast, humans appear to evaluate sequences retrospectively, often exhibiting a peak-end preference [10], [14], [29]–[34]. For example, in an experiment in which people endured the pain of placing their hands in ice-cold water [1]–[7], [34], they first experienced two options: (1) immersing one hand in 14°C water for 60 s; and (2) immersing the other hand in 14°C for 60 s then 30 additional seconds with the temperature gradually raising 15°C. When subsequently given the choice between the two options, 22 out of 32 people (69%) chose the second one even though it resulted in a 50% longer period of pain (90 s versus 60 s). The peak-end rule has been found with both positive (e.g., appetitive stimuli such as candy) and negative (e.g., aversive stimuli such as the cold water) events [10], [30], [31]. In addition, the heuristic applies not only with follow-up ratings of a previous experience, but also with subsequent choices, as in the cold-hand experiment [10], [14], [29]–[35].

Thus far, then, humans appear to exhibit a peak-end preference for outcome sequences, considering them as an episode, whereas rhesus monkeys, if anything, appear to exhibit the opposite. Whether this difference is due to species or methodological differences, however, remains unclear. For example, the human studies often use sequences composed of quantitative differences, which might be more conducive to sequence detection and formation; whereas, the rhesus study used qualitatively distinct food items. It will therefore be important to compare humans and non-human animals with more similar testing conditions, and in particular, more comparable items, which may influence sequence formation and the type of evaluation process used.

If prospective versus retrospective evaluation is at least partly based on the extent individual events are combined into sequences, one might expect self-generated sequences to promote the evaluation of each choice outcome individually, which could explain why the current results from self-generated sequences more strongly revealed a peak-first preference as opposed to the experimenter-generated sequences in the Xu et al. (2011) study. It is therefore important to ask what order humans would produce if they generated the sequences themselves. Recently, Jeong et al. (under revision) conducted such an experiment with pieces of sushi. When choosing sushi pieces without replacement, two general populations of people emerged: some people selected their preferred items first, while others saved them for last. Different subpopulations of people with opposite preferences suggest that in addition to contextual factors, individual traits may also influence the preferred sequence order. A critical individual factor might be the degree of impulsivity versus self-control, given that there are clear individual differences among humans on this trait [36]–[45].

Comparing our results with those in the sushi experiment, our rhesus monkeys match the group preferring the peak-first sushi order. Indeed, evidence points to rhesus monkeys showing relative impulsivity, compared, for example, to great apes. When required to wait for a food item, rhesus monkeys rarely exceeded a 30-second delay, whereas chimpanzees and an orangutan waited roughly six times longer [46]. In addition, rhesus monkeys appear to be significantly more sensitive to high-reward outcomes compared to low-reward outcomes: for example, (a) by being more sensitive to changes in the high-reward outcome, and (b) preferring risky options with an opportunity to receive a large outcome, compared to alternatives with equal overall payoff, but with a more evenly distributed lower reward outcome (e.g., 250 ml of fluid 50% of the time and 50 ml the other 50% of the time preferred over 150 ml 100% of the time) [47], [48].

However, it has also been argued that the apparent riskiness of rhesus monkeys may be context driven [49], reflecting the difference in experimental conditions used to study humans versus non-human animals. Evidence does suggest that this is so, with humans and other primates, for example, behaving more similarly under more similar conditions, such as people becoming riskier in a multiple trial format [50]–[54]. Nonetheless, thus far, more direct comparisons appear to show that rhesus behavior might be akin to the riskier and more impulsive human subpopulations [55], [56].

Future work will need to further delineate the environmental conditions and individual factors determining the use of prospective versus retrospective evaluation. In general, goal-directed behavior involves the generation, maintenance and seeking of goals, which appears to promote prospective evaluation [57]. Typically, when goal-seeking, it is thought to be rational to choose options with the highest immediate value, given that the risk of losing a potential outcome increases with delay. This risk is especially heightened in uncertain and competitive environments. For example, in the strict dominance hierarchies of rhesus monkeys, competition among conspecifics might in fact produce pressures to prefer the more immediately available outcomes [1]. Indeed, a great deal of evidence shows that humans and non-human animals prefer reward that arrives sooner, indicating that the value of delayed reward is discounted [58]. Moreover, the discounting of future rewards successfully explains multiple other phenomena such as preference reversals between small immediate reward and delayed larger reward, in which when a delay is long enough to both the more and less delayed rewards, one may switch to preferring the larger, more delayed reward [44], [59], [60]. In addition, there appear to be neural substrates that activate more strongly for immediate rewards, attesting to mechanisms that underlie the prospective evaluation process [44].

In more stable environments, however, sequences of events, actions and/or outcomes are more reliable, and linking (or ‘chunking’) them together into a sequence can provide efficient hierarchical memory storage and heightened predictability [61], [62]. Moreover, given that it is easier to detect a sequence after experiencing all components, and typically, after repeated exposure, this ‘chunking’ process would appear to promote retrospective evaluation [63]–[65]. It is also interesting to note the shared features of a general chunking process and retrospective evaluation with habitual behavior, which also thrives in reliable environments, strengthens with repeated exposure, and discounts events in the more distant past [61], [66].

Finally, even though humans appear to have a peak-end bias for a series of events, including, e.g., personal experiences, performances, and stories, it does not necessarily mean that if given the opportunity to build the sequence themselves, they would do so. Here we demonstrated that rhesus monkeys showed a peak-first preference in self-generated choice order. Whether self-generated sequencing imposes a heavier burden on self-control and/or sequence formation is yet to be determined. To be sure, it is likely that different underlying processes, such as prospective and retrospective evaluation, impulsivity and self-control, and the ability to detect and formulate sequences, interact to determine our preferences, and more work including comparative analyses is necessary to identify the nature of these underlying processes.
